# Increasing Taxa Sampling Provides New Insights on the Phylogenetic Relationship Between *Eriobotrya* and *Rhaphiolepis*

**DOI:** 10.3389/fgene.2022.831206

**Published:** 2022-03-15

**Authors:** Zhanghong Dong, Shaohong Qu, Sven Landrein, Wen-Bin Yu, Jing Xin, Wenzhi Zhao, Yu Song, Yunhong Tan, Peiyao Xin

**Affiliations:** ^1^ Southwest Research Center for Landscape Architecture Engineering, National Forestry and Grassland Administration, Southwest Forestry University, Kunming, China; ^2^ Center for Integrative Conservation and Horticulture Department, Xishuangbanna Tropical Botanical Garden, Chinese Academy of Sciences, Mengla, China; ^3^ Key Laboratory of Ecology of Rare and Endangered Species and Environmental Protection (Ministry of Education), Guangxi Normal University, Guilin, China; ^4^ Southeast Asia Biodiversity Research Institute, Chinese Academy of Sciences, Yezin, Myanmar

**Keywords:** *Eriobotrya*, *Rhaphiolepis*, ITS, *Maleae*, phylogenetic relationships

## Abstract

*Eriobotrya* (Rosaceae) is an economically important genus with around 30 species. It is widely distributed in tropical and warm temperate regions of Asia, with most of its species in China, Myanmar, and Vietnam. However, *Eriobotrya* is often confused with the smaller genus *Rhaphiolepis*, and the phylogenetic relationships between the two genera are controversial. Here we present phylogenetic analyses of 38 newly generated *Eriobotrya* and *Rhaphiolepis* nrDNA together with 16 sequences of nrDNA and 28 sequences of ITS obtained from GenBank, representing 28 species of *Eriobotrya* and 12 species of *Rhaphiolepis*, in order to reconstruct highly supported relationships for the two genera. Contrary to previous research based on limited sampling, our results highlight the monophyly of *Eriobotrya* as well as *Rhaphiolepis*. The topology recovered here is consistent with key morphological synapomorphies such as the persistent sepals in *Eriobotrya*. Our findings show that increased sampling of taxa can provide a more robust phylogeny through reducing phylogenetic error and increasing overall phylogenetic accuracy.

## 1 Introduction

*Eriobotrya* Lindl. and *Rhaphiolepis* Lindl., two genera of the tribe Maleae in the family Rosaceae (Kalkman, 2004), include about 30 and 15 species respectively, which are distributed throughout tropical and warm temperate regions from East Asia to tropical Southeast Asia. Loquat [*Eriobotrya japonica* (Thunb.) Lindl.] was endemic and originally domesticated in China and has been widely cultivated throughout the world. The nutritious and fleshy fruits have an attracted increasing number of consumers worldwide (Badenes et al., 2009; Blasco et al., 2014). *Rhaphiolepis indica* (L.) Lindl. also has nutritious fruits, and the red pigment in the pericarp can be used as a colorant (Huang et al., 2008).

*Eriobotrya japonica* (Thunb.) Lindl. was first described in *Flora Japonica* by Thunberg (1784) under the genus name *Mespilus* L.. John Lindley revised the genus *Mespilus* and established *Eriobotrya* as a new genus in 1882 (Lindley, 1822). *Rhaphiolepis indica* (L.) Lindl. was first described in *Species Plantarum* (1753) under the genus name *Crataegus* L.. In 1820, Lindley separated *Rhaphiolepis indica* from the genus *Crataegus* because its fruits had a papery endocarp, and he later published the genus *Rhaphiolepis* (Lindley et al., 1820). *Eriobotrya* is characterized by its fruits that have persistent sepals and leaves with excurrent lateral veins, whereas the calyx of *Rhaphiolepis* is quickly deciduous as a unit, leaving an annular ring and leaves have curved lateral veins (Vidal, 1968; Vidal, 1970; Kalkman, 1993; Lu et al., 2003; Kalkman, 2004).

It has long been difficult to classify the genera of the Maleae tribe, which may be due to polyploidy events, rapid radiations, frequent hybridizations, and/or ancient diversification among some clades (Wolfe and Wehr, 1988; Robertson et al., 1991; Vamosi and Dickinson, 2006; Campbell et al., 2007; Dickinson et al., 2007; Li et al., 2012; Lo and Donoghue, 2012; Xiang et al., 2016; Liu et al., 2019). The latest research also shows that multiple ancient hybridization and chloroplast capture events within *Eriobotrya* in the Yunnan-Guizhou Plateau (Chen et al., 2021). This makes the taxonomic study of *Eriobotrya* more complicated. In addition, the phylogenetic status of *Eriobotrya* and *Rhaphiolepis* in the Maleae tribe has always been uncertain, and the phylogenetic relationship between the two genera is also controversial. A sister relationship between *Eriobotrya* and *Rhaphiolepis* was reported for the first time by Campbell et al. (1995) using the nuclear ribosomal internal transcribed spacer (nrITS) (Campbell et al., 1995). Six chloroplast DNA (cpDNA) regions, two GBSSI genes (1A and 2B), and nrITS sequences supported a sister relationship between *Eriobotrya* and *Rhaphiolepis* (Campbell et al., 2007). Phylogenetic relationships among 88 genera of Rosaceae were investigated using nucleotide sequence data from six nuclear and four chloroplast regions, and the results showed that *Eriobotrya* and *Rhaphiolepis* were sister groups (Li et al., 2012). In addition, nrITS data supported the monophyly of *Eriobotrya*, with *Rhaphiolepis indica* as a sister to the *Eriobotrya* clade (Li et al., 2012). Further studies in Maleae, also showed this sister relationship was supported using chloroplast, nrITS, and even whole plastome sequences (Lo and Donoghue, 2012; Xiang et al., 2016; Zhang et al., 2017; Sun et al., 2018; Liu et al., 2019; Idrees et al., 2020b). In a preliminarily phylogenic study of the *Eriobotrya* genus based on the nuclear ribosomal DNA (nrDNA) *Adh* sequences, *R. indica* was shown to be sister to a subclade of *Eriobotrya* and they suggested paraphyly (Yang et al., 2012). Close morphological and genetic relationships have been found in almost all studies involving *Eriobotrya* and *Rhaphiolepis*, there have been very few cases showing paraphyly and there was no case for merging them, despite the existence of intergeneric hybrids (Aldasoro et al., 2005; Sun et al., 2018).

A present genomic study, however supports incorporating *Eriobotrya* into the *Rhaphiolepis* genus and renaming all species within the *Eriobotrya* genus (Liu et al., 2020). In that research analyzed 16 nrDNA sequences and 21 complete plastomes indicating that the *Rhaphiolepis* species were nested among the *Eriobotrya* taxa (Liu et al., 2020). Morphologically, the persistent sepals and the excurrent lateral veins of the leaves are used to distinguish the two genera (Vidal, 1968; Vidal, 1970; Kalkman, 1993; Lu et al., 2003; Kalkman, 2004), but researchers have found that the sepals of *Eriobotrya henryi* Nakai are obviously caducous, the lateral veins of the leaves in *E. henryi* and *Eriobotrya seguinii* J. E. Vidal are curved, and the lateral veins of *Rhaphiolepis ferruginea* F. P. Metcalf sometimes terminate at the leaf margins (Liu et al., 2020). Furthermore, the seeds of the two genera are rounded or widely elliptic with an absence of endosperm (Liu et al., 2020). Geographically, these two genera overlap broadly in East and Southeast Asia (Liu et al., 2020). However, the latest research (Kang et al., 2021) does not support the results of Liu et al. These latter results show that Bayesian inference (BI) and maximum likelihood (ML) trees exhibit a well-supported monophyly for *Eriobotrya*, which is separate and distinct from *Rhaphiolepis* (Kang et al., 2021). To better estimate the phylogenetic relationship between *Eriobotrya* and *Rhaphiolepis*, it is necessary to sample more taxa to reliably reconstruct the phylogenetics of the *Eriobotrya* and *Rhaphiolepis* genera.

## 2 Materials and Methods

### 2.1 DNA Extraction and Sequencing

**FIGURE 1:**
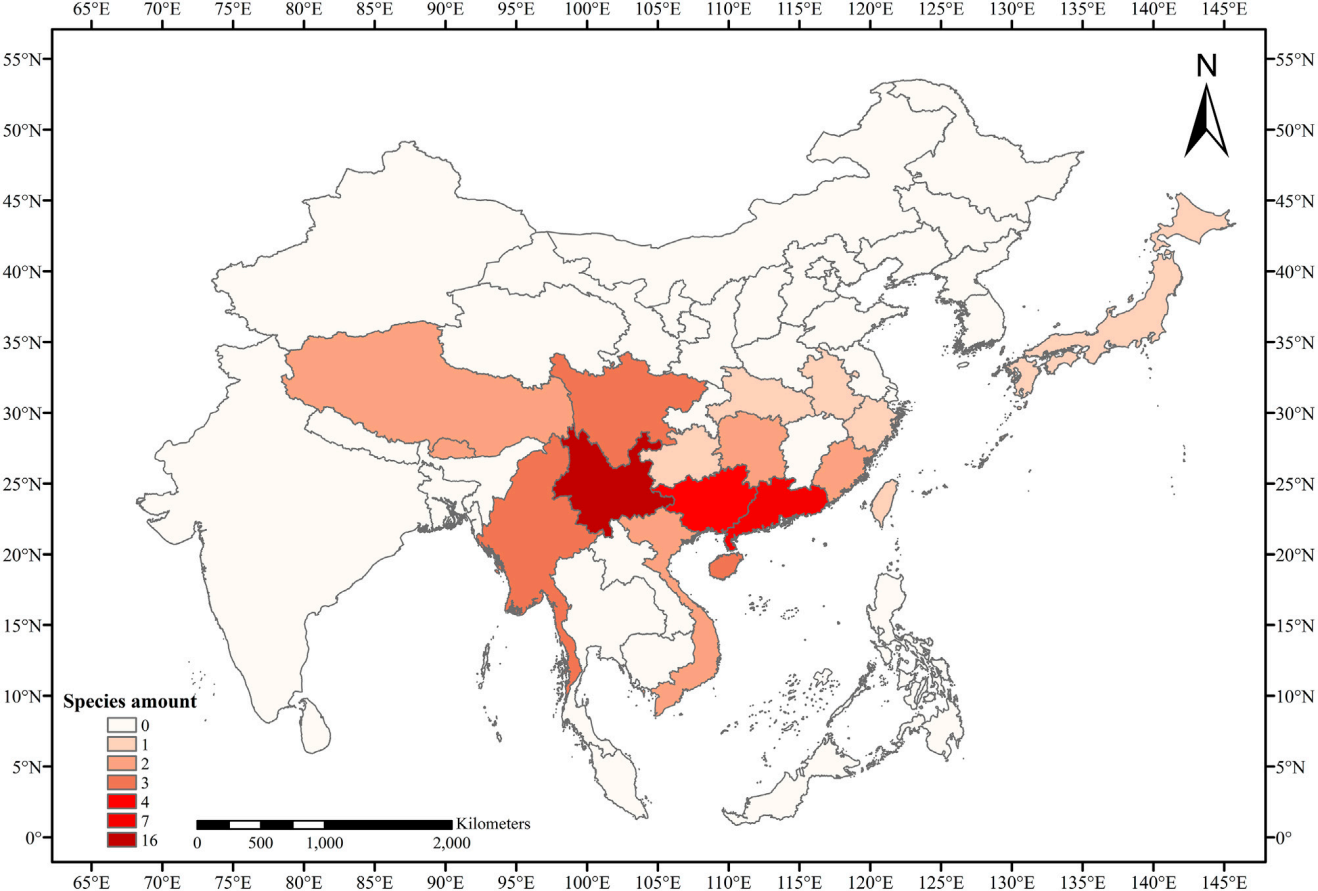
Species diversity heat map of *Eriobotrya* and *Rhaphiolepis* in this study.

Thirty-eight taxa from *Eriobotrya* and *Rhaphiolepis* were collected, with additional 16 nrDNA and 28 nrITS sequences from GenBank added (Table 1, and Supplementary Table S1). The species of *Eriobotrya* and *Rhaphiolepis* collected covered all main plant-distribution regions (Figure 1). Genomic DNA was extracted from 3 g of fresh leaves or from silica-dried leaf materials using the modified cetyltrimethylammonium bromide (CTAB) method (Liu et al., 2005), in which 4% CTAB was used, and we added ∼1% polyvinyl polypyrrolidone (PVP) and 0.2% DL-dithiothreitol (DTT). The nrDNA was sequenced following Zhang et al. (2016), and the long-range PCR was used for next-generation sequencing with a primer pair for nuclear ribosomal DNA (rRNA_2F: TAA​GCC​ATG​CAT​GTG​TAA​GTA​TGA​AC; rRNA_2R: CGT​ATT​TAA​GTC​GTC​TGC​AAA​GGA​TT). Sequencing was performed at Annoroad Gene Technology Co., Ltd., Beijing, China.

**TABLE 1:** List of samples, voucher collection information, and accession numbers.

No	Taxa	Herbarium	Voucher	Geographic origin	Accession no
1	*Eriobotrya* × *daduheensis* H.Z. Zhang ex W.B. Liao, Q. Fan & M.Y. Ding	HITBC-BRG	SY36983	Sichuan, China	RHA10032
2	*Eriobotrya bengalensis* (Roxb.) Kurz	HITBC-BRG	SY35370	Yunnan, China	RHA10003
3	*Eriobotrya bengalensis* (Roxb.) Kurz	HITBC-BRG	SY35363	Yunnan, China	RHA10020
4	*Eriobotrya bengalensis* var. *angustifolia* Cardot	HITBC-BRG	SY34843	Yunnan, China	RHA10008
5	*Eriobotrya deflexa* (Hemsl.) Nakai	HITBC-BRG	SY34244	Hainan, China	RHA10012
6	*Eriobotrya fragrans* Champ. ex Benth	HITBC-BRG	SY35360	Yunnan, China	RHA10001
7	*Eriobotrya glabrescens* J.E. Vidal	HITBC-BRG	M1286	Kachin State, Myanmar	RHA10036
8	*Eriobotrya glabrescens* J.E. Vidal	HITBC-BRG	M1248	Kachin State, Myanmar	RHA10037
9	*Eriobotrya glabrescens* J.E. Vidal	HITBC-BRG	M1210	Kachin State, Myanmar	RHA10038
10	*Eriobotrya henryi* Nakai	HITBC-BRG	SY35054	Yunnan, China	RHA10005
11	*Eriobotrya henryi* Nakai	HITBC-BRG	SY33356	Yunnan, China	RHA10006
12	*Eriobotrya henryi* Nakai	HITBC-BRG	SY36653	Yunnan, China	RHA10007
13	*Eriobotrya japonica* (Thunb.) Lindl	HITBC-BRG	SY36504	Tibet, China	RHA10013
14	*Eriobotrya japonica* (Thunb.) Lindl	HITBC-BRG	SY36989	Yunnan, China	RHA10014
15	*Eriobotrya japonica* (Thunb.) Lindl	HITBC-BRG	SY36988	Sichuan, China	RHA10015
16	*Eriobotrya japonica* (Thunb.) Lindl	HITBC-BRG	SY35404	Yunnan, China	RHA10019
17	*Eriobotrya laoshanica* W.B. Liao, Q. Fan & S.F. Chen	HITBC-BRG	SY36641	Yunnan, China	RHA10024
18	*Eriobotrya laoshanica* W.B. Liao, Q. Fan & S.F. Chen	HITBC-BRG	SY36731	Yunnan, China	RHA10026
19	*Eriobotrya malipoensis* K.C. Kuan	HITBC-BRG	SY35055	Yunnan, China	RHA10002
20	*Eriobotrya malipoensis* K.C. Kuan	HITBC-BRG	SY36710	Yunnan, China	RHA10021
21	*Eriobotrya platyphylla* Merr	HITBC-BRG	M2963	Kachin State, Myanmar	RHA10035
22	*Eriobotrya prinoides* Rehder & E.H. Wilson	HITBC-BRG	SY33357	Yunnan, China	RHA10022
23	*Eriobotrya prinoides* Rehder & E.H. Wilson	HITBC-BRG	SY36984	Sichuan, China	RHA10023
24	*Eriobotrya serrata* J.E. Vidal	HITBC-BRG	SY36349	Yunnan, China	RHA10034
25	*Eriobotrya serrata* J.E. Vidal	HITBC-BRG	SY36349	Yunnan, China	RHA10033
26	*Eriobotrya* sp1	HITBC-BRG	SY34277	Yunnan, China	RHA10010
27	*Eriobotrya* sp1	HITBC-BRG	SY36696	Yunnan, China	RHA10011
28	*Eriobotrya* sp2	HITBC-BRG	SY36770	Yunnan, China	RHA10027
29	*Eriobotrya* sp3	HITBC-BRG	SY35312	Yunnan, China	RHA10028
30	*Eriobotrya* sp4	HITBC-BRG	M6196	Kachin State, Myanmar	RHA10047
31	*Eriobotrya* sp5	HITBC-BRG	SY34776	Yunnan, China	RHA10029
32	*Eriobotrya* sp5	HITBC-BRG	SY36358	Yunnan, China	RHA10030
33	*Eriobotrya* sp5	HITBC-BRG	SY36410	Yunnan, China	RHA10031
34	*Eriobotrya tengyuehensis* W. W. Smith	HITBC-BRG	SY36962	Yunnan, China	RHA10041
35	*Rhaphiolepis brevipetiolata* J.E. Vidal	HITBC-BRG	SY36976	Yunnan, China	RHA10043
36	*Rhaphiolepis indica* var. *tashiroi* Hayata ex Matsum. & Hayata	HITBC-BRG	SY36977	Yunnan, China	RHA10044
37	*Rhaphiolepis mekongensis* (Cardot) Tagane & H. Toyama	HITBC-BRG	SY36974	Yunnan, China	RHA10042
38	*Rhaphiolepis* sp	HITBC-BRG	SY34214	Hainan, China	RHA10009

### 2.2 Nuclear Ribosomal DNA Assembly and Annotation

Paired-end reads were filtered with the GetOrganelle software, using the following parameters for word sizes, rounds, k-mer, and pregouping: -w 103, -R 10, -K 75 to 105, -P 300,000, respectively (Jin et al., 2020). *Eriobotrya cavaleriei* (H. Léveillé) Rehder (GenBank accession number: MN215982) was chosen as a reference, and the nrDNA sequences were adjusted and annotated with Geneious 8.1.3 software (Kearse et al., 2012). Correlations among these parameters were explored by employing Pearson Correlation Coefficient reporting and *r* ^*2*^ *-values*. The annotated nrDNA sequences were submitted to the Rosaceae Chloroplast Genome Database (https://lcgdb.wordpress.com/category/rosaceae/) (Table 1).

### 2.3 Mutation Events Analysis

To identify the microstructural mutations between *Eriobotrya* and *Rhaphiolepis*, the two genera-aligned sequences were further analyzed with MAFFT version 7 software (Katoh and Standley, 2013) and then manually aligned with MEGA X (Kumar et al., 2018). Indel and single-nucleotide polymorphism (SNP) events were counted and positioned in the nrDNA using manual statistics. We also conducted a sliding-window analysis to evaluate the nucleotide variability (Pi) throughout the nrDNA in DnaSP version 6 software (Rozas et al., 2017). The window length was set to 100 bp and the step size to 50 bp.

### 2.4 Phylogenetic Analyses

Matrix I included 97 nrDNA sequences, including *Chaenomeles* Lindl. (2 spp.), *Cydonia* Mill. (1 sp.), *Dichotomanthes* Kurz. (1 sp.), *Docynia* Decne. (1 sp.), *Eriobotrya* Lindl. (23 spp.), *Heteromeles* M. Roem (1 sp.), *Malus* Mill. (6 spp.), *Phippsiomeles* B.B. Liu & J. Wen (3 spp.), *Photinia* Lindl. (13 spp.), *Pourthiaea* Decne. (6 spp.), *Pyracantha* M. Roem. (2 spp.), *Pyrus* L. (1 sp.), *Rhaphiolepis* Lindl. (11 spp.), and *Stranvaesia* Lindl. (3 spp.). *Gillenia trifoliata* (L.) Moench and *Gillenia stipulata* (Muhl.ex Willd.) Nutt. were used as outgroups (Supplementary Table S2). Matrix II included 42 *Eriobotrya* Lindl., *Rhaphiolepis* Lindl., and *Phippsiomeles* B.B. Liu & J. Wen species, including 57 nrDNA sequences and 29 additional taxa with nrITS sequences (ITS1, 5.8S, and ITS2). *Phippsiomeles matudae* (Lundell) B.B. Liu & J. Wen, *Phippsiomeles mexicana* (Baill.) B.B. Liu & J. Wen, and *Phippsiomeles microcarpa* (Standl.) B. B. Liu & J. Wen were used as outgroups (Supplementary Table S3). To evaluate potential conflict among regions, we divided matrix I into nine subsets: ETS, ITS1, ITS2, ETS–ITS1, ETS–ITS2, ITS1–ITS2, 26S, and 18S–5.8S–26S. The sequence matrix was aligned with MAFFT version 7 software (Katoh and Standley, 2013) and then manually edited with MEGA X (Kumar et al., 2018).

Two different data matrices were aligned and analyzed using BI, ML, and Parsimony (P) methods. The BI was performed with BEAST version 2.6.3 (Bouckaert et al., 2019), using the best-fit DNA replacement model selected by jModelTest 2.1.10 for the phylogenetic reconstruction (Darriba et al., 2012). The Markov chain Monte Carlo (MCMC) algorithm was run for 100,000,000 generations, and the BI analysis started with a random tree and was sampled every 1,000 generations. The first 20% of the tree was discarded as burn-in, and the remaining tree was used to produce a majority-rule consensus tree. The ML analysis was carried out with IQ-TREE version 1.6.7 (Nguyen et al., 2015) with 1,000 bootstrap(BS) replicates using UFBoot2 (Hoang et al., 2018) and collapsing the near zero branches option. The P analysis was performed with MEGA X (Kumar et al., 2018) and 1,000 BS replicates.

## 3 Results

### 3.1 Size and Organization of the nrDNA Sequences

The size of the 38 newly determined *Eriobotrya* and *Rhaphiolepis* nrDNA sequences ranged from 6,775 bp (*Eriobotrya glabrescens* J.E. Vidal) to 6,803 bp (*E. henryi*) (Table 2). These nrDNA sequences included three rRNA genes and three transcribed spacers. In the 26S large-subunit rRNA (26S) region, the length varied from 3,358 bp (*E. glabrescens*) to 3,387 bp (*E. henryi*); in the 18S small-subunit rRNA (18S) region, 1,808 bp; in the 5.8S rRNA (5.8S) region, 159 bp; in the external transcribed spacer (ETS) region, from 1,016 to 1,019 bp, in the ITS1 region from 217 to 219 bp; and in the ITS2 region from 207 to 217 bp. The overall G + C content was 56.0–56.5%. The G + C content of the rRNA region varied from 55.0 to 55.3% (18S, 49.8–49.9%; 5.8S, 56.6–57.9%; 26S, 57.7–58.1%), and that of gene spacer region varied from 59.5 to 60.8% (ETS, 56.3–57.6%; ITS1, 64.1–66.4%; ITS2, 67.4–72.1%). Correlations were tested between the size of the nrDNA sequence and each of the six regions and the GC content of the nrDNA sequence and each of the six regions. The *r* ^*2*^ and Pearson results (*r* ^*2*^ > 0.5 and *p* < 0.05) were considered significant. There were significant correlations in the sequence size between nrDNA and 26S and in the GC content between nrDNA and ETS, ITS2, and 26S (Table 3).

**TABLE 2:** Summary of 38 nrDNA of *Eriobotrya* and *Rhaphiolepis*.

Taxon	Accession no	Total nrDNA size(bp)	Total GC content (%)	ETS size (bp)	ETS GC content (%)	18S (bp)	18S GC content (%)	ITS1 size (bp)	ITS1 GC content (%)	5.8S size (bp)	5.8 GC content (%)	ITS2 size (bp)	ITS2 GC content (%)	26S size (bp)	26S GC content (%)
*E. × daduheensis*	RHA10032	6,788	56.20	1,016	56.80	1,808	49.80	217	65.00	159	57.90	217	70.00	3,371	58.00
*E. bengalensis*	RHA10020	6,788	56.30	1,016	56.90	1,808	49.80	217	65.00	159	57.90	217	71.40	3,371	58.10
*E. bengalensis*	RHA10003	6,788	56.40	1,016	57.00	1,808	49.80	217	65.00	159	57.90	217	71.40	3,371	58.10
*E. bengalensis* var. *angustifolia*	RHA10008	6,788	56.00	1,016	56.60	1,808	49.80	217	65.00	159	57.20	217	68.20	3,371	57.80
*E. deflexa*	RHA10012	6,786	56.10	1,017	56.80	1,808	49.80	219	65.80	159	57.90	215	70.20	3,368	57.70
*E. fragrans*	RHA10001	6,788	56.10	1,017	56.30	1,808	49.90	217	65.00	159	57.20	215	68.80	3,372	57.90
*E. glabrescens*	RHA10036	6,775	56.00	1,016	56.50	1,808	49.80	217	64.50	159	57.20	217	69.60	3,358	57.80
*E. glabrescens*	RHA10037	6,788	56.10	1,016	56.50	1,808	49.80	217	64.50	159	57.20	217	69.60	3,371	57.90
*E. glabrescens*	RHA10038	6,788	56.10	1,016	56.50	1,808	49.80	217	64.50	159	57.20	217	69.60	3,371	57.90
*E. henryi*	RHA10006	6,778	56.30	1,016	57.60	1,808	49.90	217	64.10	159	57.20	207	71.50	3,371	57.80
*E. henryi*	RHA10007	6,787	56.30	1,016	57.60	1,808	49.90	218	64.20	159	57.20	215	72.10	3,371	57.80
*E. henryi*	RHA10005	6,803	56.30	1,016	57.60	1,808	49.90	218	64.20	159	57.20	215	72.10	3,387	57.80
*E. japonica*	RHA10015	6,786	56.10	1,016	56.90	1,808	49.80	217	65.00	159	56.60	215	69.30	3,371	57.90
*E. japonica*	RHA10019	6,786	56.10	1,016	56.90	1,808	49.80	217	65.00	159	56.60	215	69.30	3,371	57.90
*E. japonica*	RHA10013	6,786	56.10	1,016	56.90	1,808	49.80	217	65.00	159	56.60	215	69.50	3,371	57.90
*E. japonica*	RHA10014	6,786	56.20	1,016	56.90	1,808	49.80	217	65.00	159	56.60	215	69.80	3,371	57.90
*E. laoshanica*	RHA10024	6,788	56.00	1,018	56.60	1,808	49.90	217	65.00	159	56.60	215	67.90	3,371	57.70
*E. laoshanica*	RHA10026	6,789	56.10	1,019	56.80	1,808	49.90	217	65.00	159	56.60	215	69.30	3,371	57.70
*E. malipoensis*	RHA10002	6,786	56.20	1,016	56.90	1,808	49.80	217	65.00	159	57.20	215	69.80	3,371	57.90
*E. malipoensis*	RHA10021	6,786	56.20	1,016	56.90	1,808	49.80	217	65.40	159	57.20	215	69.80	3,371	58.00
*E. platyphylla*	RHA10035	6,788	56.20	1,016	56.60	1,808	49.90	217	65.00	159	57.90	217	70.50	3,371	58.00
*E. prinoides*	RHA10022	6,788	56.10	1,016	56.60	1,808	49.90	217	64.50	159	57.90	217	69.60	3,371	57.80
*E. prinoides*	RHA10023	6,788	56.20	1,016	56.70	1,808	49.90	217	64.50	159	57.90	217	69.60	3,371	57.90
*E. serrata*	RHA10034	6,787	56.10	1,016	56.70	1,808	49.80	217	65.90	159	57.20	215	67.40	3,372	57.90
*E. serrata*	RHA10033	6,788	56.10	1,017	56.60	1,808	49.80	217	65.90	159	57.20	215	67.90	3,372	57.90
*E. tengyuehensis*	RHA10041	6,788	56.00	1,016	56.40	1,808	49.80	217	65.00	159	57.20	217	69.10	3,371	57.80
*E.* sp1	RHA10010	6,788	56.20	1,016	56.50	1,808	49.80	217	65.90	159	57.20	217	70.00	3,371	58.00
*E.* sp1	RHA10011	6,788	56.20	1,016	56.50	1,808	49.80	217	65.90	159	57.20	217	70.00	3,371	58.00
*E.* sp2	RHA10027	6,786	56.10	1,016	56.80	1,808	49.80	217	64.50	159	56.60	215	69.30	3,371	57.90
*E.* sp3	RHA10028	6,788	56.00	1,016	56.50	1,808	49.80	217	64.50	159	57.20	217	69.10	3,371	57.80
*E.* sp4	RHA10047	6,788	56.20	1,016	56.80	1,808	49.90	217	64.50	159	57.90	217	70.00	3,371	57.90
*E.* sp5	RHA10031	6,780	56.20	1,016	56.60	1,808	49.80	217	65.40	159	57.20	217	70.50	3,363	57.90
*E.* sp5	RHA10029	6,788	56.20	1,016	56.50	1,808	49.80	217	65.40	159	57.20	217	70.50	3,371	57.90
*E.* sp5	RHA10030	6,788	56.20	1,016	56.70	1,808	49.80	217	65.40	159	57.20	217	70.50	3,371	57.90
*R. brevipetiolata*	RHA10043	6,786	56.50	1,016	57.40	1,808	49.90	217	65.90	159	57.20	215	71.20	3,371	58.10
*R. indica* var. *tashiroi*	RHA10044	6,786	56.50	1,016	57.40	1,808	49.90	217	65.90	159	57.20	215	70.70	3,371	58.10
*R. mekongensis*	RHA10042	6,786	56.50	1,016	57.40	1,808	49.90	217	65.90	159	57.20	215	71.20	3,371	58.10
*R.* sp	RHA10009	6,786	56.50	1,016	57.40	1,808	49.90	217	66.40	159	57.20	215	71.20	3,371	58.10

**TABLE 3:** Correlations between main characteristics of *Eriobotrya* and *Rhaphiolepis* nrDNA.

p/*r* ^2^	nrDNA	GC	ETS	ETS GC	18S GC	ITS1	ITS1 GC	5.8S GC	ITS2	ITS2 GC	26S	26 SGC
nrDNA	–	0.0032	0.0115	0.0063	0.0293	0.0650	0.0043	0.0084	0.0714	0.0058	**0.7904**	0.0005
GC	0.7360	–	0.0775	**0.5727**	0.1970	0.0004	0.1401	0.0528	0.0508	**0.5848**	0.0455	**0.5329**
ETS	0.5220	0.0910	–	0.0270	0.0736	0.0168	0.0049	0.0736	0.0210	0.1204	0.0000	0.2286
ETS GC	0.6370	**0.0000**	0.3250	–	0.2378	0.0821	0.0001	0.0031	0.3423	0.4764	0.1314	0.0694
18S GC	0.3040	0.0050	0.0990	0.0020	–	0.0056	0.0134	0.0208	0.1043	0.1085	0.0802	0.0000
ITS1	0.1220	0.9030	0.4380	0.0810	0.6550	–	0.0020	0.0044	0.0135	0.0928	0.0397	0.1466
ITS1GC	0.6970	0.0210	0.6760	0.9470	0.4880	0.7880	–	0.0013	0.0086	0.0053	0.0146	0.2538
5.8S GC	0.5840	0.1650	0.0990	0.7420	0.3880	0.2060	0.8310	–	0.1043	0.0978	0.0011	0.0618
ITS2	0.1050	0.1740	0.3850	0.0000	0.0480	0.4870	0.5790	0.0480	–	0.0262	0.0254	0.0247
ITS2 GC	0.6500	**0.0000**	0.0330	0.0000	0.0430	0.0630	0.6640	0.0560	0.3310	–	0.0342	0.1553
26S	**0.0000**	0.1990	0.9970	0.0250	0.0850	0.2300	0.4690	0.8400	0.3390	0.2660	–	0.0005
26S GC	0.8930	**0.0000**	0.0020	0.1100	0.9760	0.0180	0.0010	0.1320	0.3460	0.0140	0.8970	–

*r* ^2^ > 0.5, *p < 0.01* to show correlation.

### 3.2 Numbers and Pattern of Indel and Single-Nucleotide Polymorphism Mutations in nrDNA Sequences

To detect variable sites in the nrDNA of *Eriobotrya* and *Rhaphiolepis*, indel mutations among the 38 sequences were identified. A total of 21 indels were detected in the 38 sequences, including seven indels in the 26S region, six indels in the ETS region, four indels in the ITS2 region, and four indels in the ITS1 region (Table 4). All 21 indels occurred in the nrDNA sequences of the *Eriobotrya* taxa, rather than that of the *Rhaphiolepis* taxa.

**TABLE 4:** Forms and numbers of indel mutational events in the nrDNA between the genera of *Eriobotrya* and *Rhaphiolepis.*

No	Location	Motif	Size	Driection^a^	Type
1	EST	CG	2	Insertion	non-SSR
2	EST	T	1	Insertion	SSR
3	EST	G/T	1	Deletion	non-SSR
4	EST	T	1	Insertion	SSR
5	EST	A	1	Insertion	SSR
6	EST	T	1	Insertion	SSR
7	ITS1	G	1	Insertion	SSR
8	ITS1	A	1	Insertion	SSR
9	ITS1	A	1	Insertion	SSR
10	ITS1	G	1	Insertion	SSR
11	ITS2	T/C	1	Deletion	non-SSR
12	ITS2	CG	2	Insertion	SSR
13	ITS2	GTGCGTCG	8	Deletion	non-SSR
14	ITS2	A	1	Insertion	non-SSR
15	26S	CCGGGCTGTTGGTATG	16	Insertion	non-SSR
16	26S	GCGGAGACGCCGT	13	Deletion	non-SSR
17	26S	TGGCGGGCA	9	Deletion	non-SSR
18	26S	C	1	Insertion	SSR
19	26S	G	1	Insertion	SSR
20	26S	T	1	Insertion	SSR
21	26S	A	1	Insertion	SSR

a Reference to the nrDNA, sequence of *Eriobotrya japonica* (RHA10014).

The SNP markers were also counted in the nrDNA sequences of *Eriobotrya* and *Rhaphiolepis* species. The nrDNA of *E. japonica* (RHA10014) was used as a reference. We detected a total of 348 SNPs (Supplementary Table S4), including 258 transitions (Ts) and 90 transversions (Tv) (Supplementary Figure S1). The Ts-to-Tv ratio was 1:0.35. Among the Tv, 10 were Tv between the T and the A, 23 were Tv between the C and the G, and the other 315 were related to GC content changes. In the rRNA gene regions, we detected 108 SNPs, including 94 SNPs in the 26S region, 11 SNPs in the 18S region, and three SNPs in the 5.8S region. In the gene spacer regions, we detected 240 SNPs, including 151 SNPs in the ETS region, 61 SNPs in the ITS2 region, and 28 SNPs in the ITS1 region. These 348 SNPs in two genera, included 197 SNPs that occurred only in the *Eriobotrya* species, accounting for 56.61% of all SNPs; 84 SNPs occurred only in the *Rhaphiolepis* species, accounting for about 24.14% of all SNPs, and 67 SNPs occurred in the species of both genera, accounting for about 19.25% of all SNPs (Supplementary Table S4 and Supplementary Figure S2).

**FIGURE 2:**
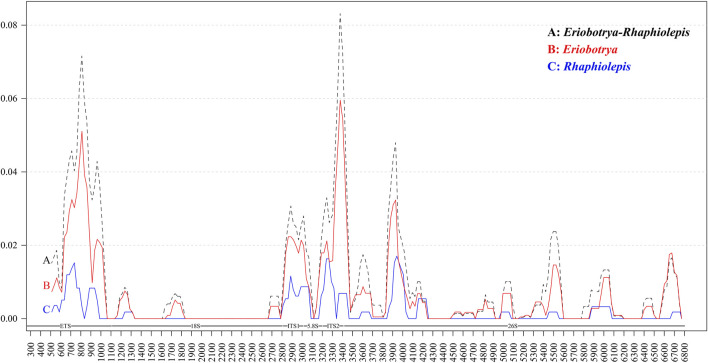
Sliding window analysis of the nrDNA of *Eriobotrya* and *Rhaphiolepis* **(A)**, *Eriobotrya* **(B)**, and *Rhaphiolepis* **(C)**. (window length: 100 bp, step size: 50 bp). *x* axis, position of the midpoint of the window; *y* axis, nucleotide diversity of each window.

To elucidate the level of sequence divergence, the Pi values within 100 bp in the nrDNA of both *Eriobotrya* and *Rhaphiolepis*, were calculated with DnaSP 6.0 software (Figure 2). Within the combined *Eriobotrya* and *Rhaphiolepis* genera, those values varied from 0 to 0.08315, with a mean of 0.01988 (Figure 2A). Within *Eriobotrya*, those values varied from 0 to 0.05959, with a mean of 0.00665 (Figure 2B). Within the *Rhaphiolepis*, those values varied from 0 to 0.01709, with a mean of 0.001812 (Figure 2C). The results show that the differences between the two genera were larger than those among congeneric species. Three regions including ETS, ITS1, and ITS2 were particularly highly variable between *Eriobotrya* and *Rhaphiolepis* and among the congeneric species.

### 3.3 Phylogenetic Analyses Based on nrDNA and ITS Region

**FIGURE 3:**
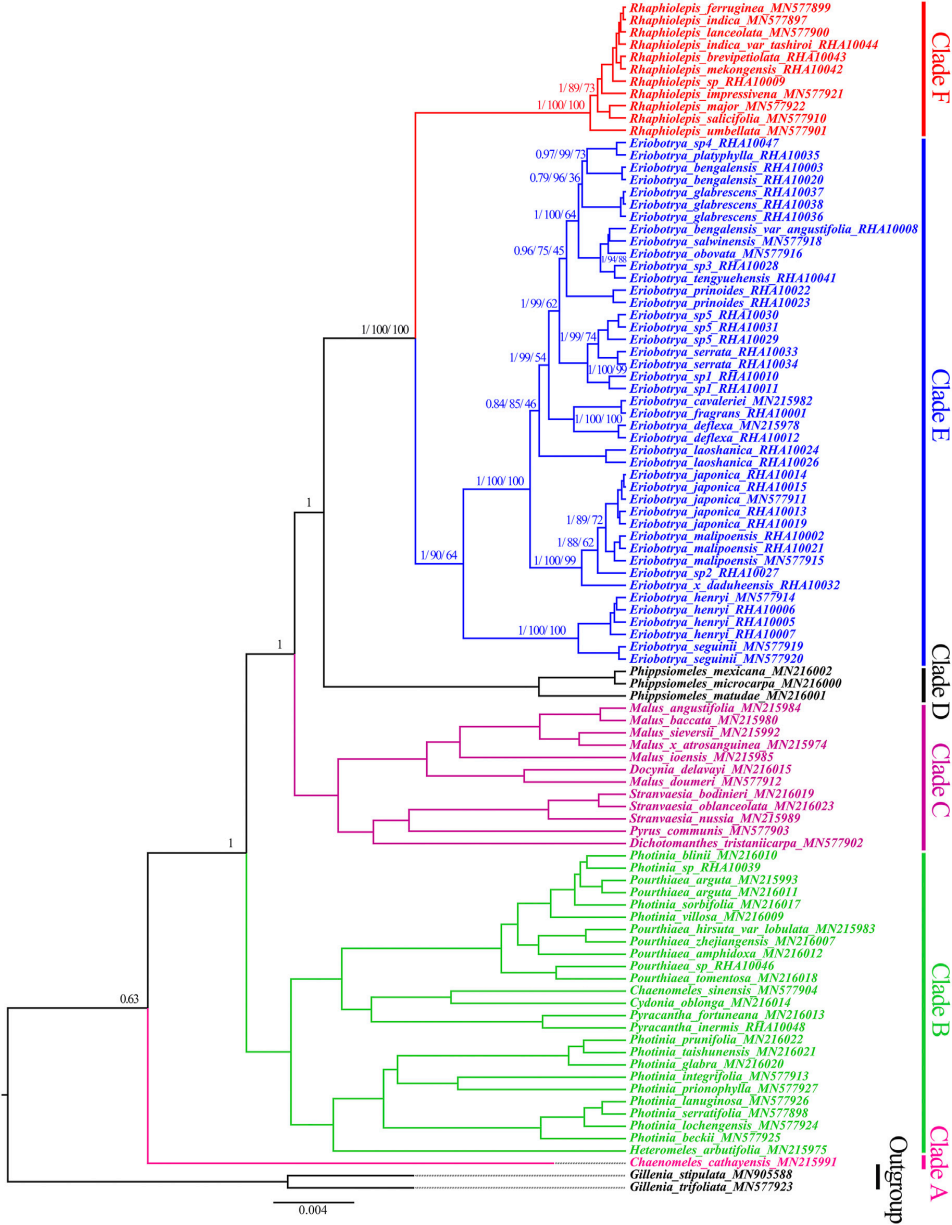
Molecular phylogenetic tree of 97 taxa of Rosaceae based on nrDNA sequences using Bayesian inference. Numbers at each node are the Bayesian posterior probabilities/maximum likelihood bootstrap support/maximum parsimony bootstrap support values. Different branches are marked as Clade A, Clade B, Clade C, Clade D, Clade E, and Clade F. The tree was rooted using the nrDNA sequence of *Gillenia trifoliate* and *G. stipulate* as outgroups.

BI, ML, and P analyses of the nrDNA sequence fully resolved the phylogenetic relationships among the *Eriobotrya* and *Rhaphiolepis* species, and most resolved relationships had high internal support (Figure 3, Supplementary Figures S3, S4). In the BI, and ML, and P trees of the nrDNA, the *Rhaphiolepis* was strongly supported as monophyletic (BI, posterior probability [PP] = 1.00, ML–BS = 100%, and P–BS = 100%), the *Eriobotrya* was also strongly supported as monophyletic (BI–PP = 1.00, ML–BS = 90%, and P–BS = 64%), and sisterhood of *Rhaphiolepis* and *Eriobotrya* was highly supported (BI–PP = 1.00, ML–BS = 100%, and P–BS = 100%). In the BI tree, the first clade (BI–PP = 0.63) included one species of *Chaenomeles cathayensis* (Hemsl.) C. K. Schneid (Clade A); the second clade (BI–PP = 1.00) included species of *Heteromeles*, *Photinia* Lindl., *Pyracantha* M. Roem., *Cydonia* Mill., *Chaenomeles* Lindl., and *Pourthiaea* Decne.; the third clade (BI–PP = 1.00) included species of *Dichotomanthes* Kurz., *Pyrus* Linnaeus., S*tranvaesia* Lindl., *Malus* Mill., and *Docynia* Decne.; the fourth clade (BI–PP = 1.00) included species of *Phippsiomeles*; the fifth clade (BI–PP = 1.00, ML–BS = 100%, P–BS = 100%) included species of *Eriobotrya*; and the sixth clade (BI–PP = 1.00 ML–BS = 100%, and MP–BS = 100%) included species of *Rhaphiolepis* (Figure 3).

**FIGURE 4:**
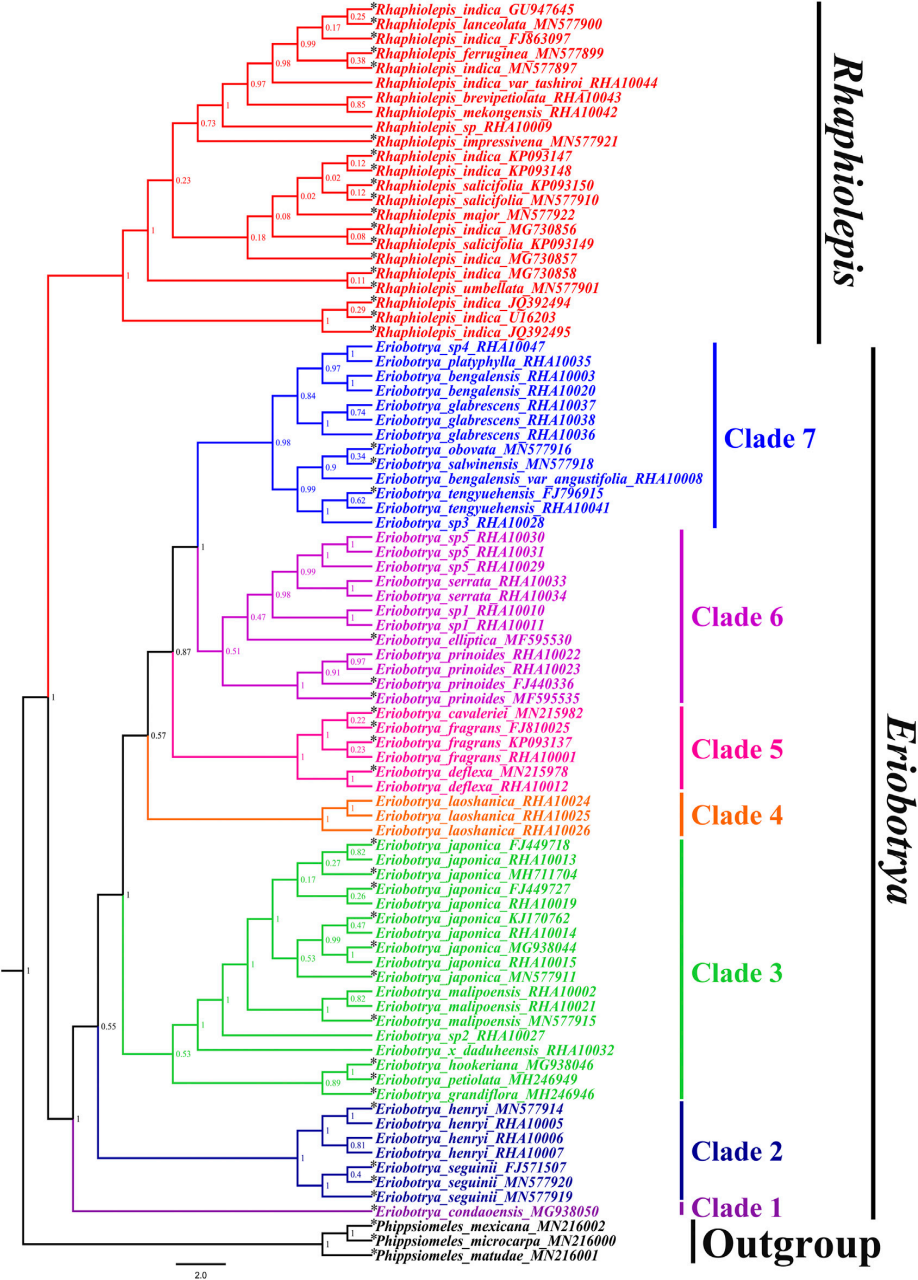
Molecular phylogenetic tree of 86 taxa of *Eriobotrya* and *Rhaphiolepis* and three related taxa of *Phippsiomeles*, based on the nrDNA sequence and ITS (containing only ITS1, 5.8S, and ITS2) sequences using Bayesian inference. Numbers at each node are Bayesian posterior probabilities. The tree is rooted with the nrDNA sequences of *Phippsiomeles matudae*, *Phippsiomeles mexicana*, and *Phippsiomeles microcarpa*. The asterisks (*) indicate the sampling in NCBI.

To better understand the phylogenetic relationships among the sequenced taxa from *Eriobotrya* and *Rhaphiolepis*, we downloaded available ITS sequences from GenBank, including 27 *Eriobotrya* and *Rhaphiolepis* taxa. *Phippsiomeles matudae*, *P. mexicana*, and *P. microcarpa* were used as outgroups. Both BI and ML trees supported sisterhood between *Eriobotrya* and *Rhaphiolepis* (Figure 4, and Supplementary Figure S5). According to the BI tree, *Eriobotrya* can be divided into seven clades. Clade 1 (BI–PP = 1.00) included one species from Vietnam: *E. condaoensis* X.F. Gao, Idrees & T.V. Do. Clade 2 (BI–PP = 0.55) included two species: *E. seguinii* and *E. henryi*. Clade 3 (BI–PP = 1.00) included six species: *E. grandiflora* Rehder & E.H. Wilson, *E. petiolata* Hook. f, *E. hookeriana* Decne, *E.* × *daduheensis* H.Z. Zhang ex W.B. Liao, *E.* sp2, *E. malipoensis* Kuan, and *E. japonica* (Thunb.) Lindl. Clade 4 (BI–PP = 0.57) included one species hfrom Vietnam and Yunan: *E. laoshanica* W.B. Liao. Clade 5 (BI–PP = 0.87) included three species: *E. deflexa* (Hemsl.) Nakai, *E. fragrans* Champ, and *E. cavaleriei* (Levl.) Rehd. Clade 6 (BI–PP = 1) included five species: *E. prinoides* Rehd. et Wils, *E. elliptica* Lindley, *E.* sp1, *E. serrata* Vidal, and *E.* sp5. Clade 7 (BI–PP = 1) included nine species: *E.* sp3, *E. tengyuehensis* W.W. Smith, *E. bengalensis* var. *angustifolia* Cardot, *E. salwinensis* Hand.-Mazz, and *E. obovata* W.W. Smith, *E. glabrescens* J.E. Vidal, *E. bengalensis* (Roxb.) Hook. f, *E. platyphylla* Merr, and *E.* sp4.

### 3.4 Phylogenetic Analyses Based on Six Regions of nrDNA Sequences

**FIGURE 5:**
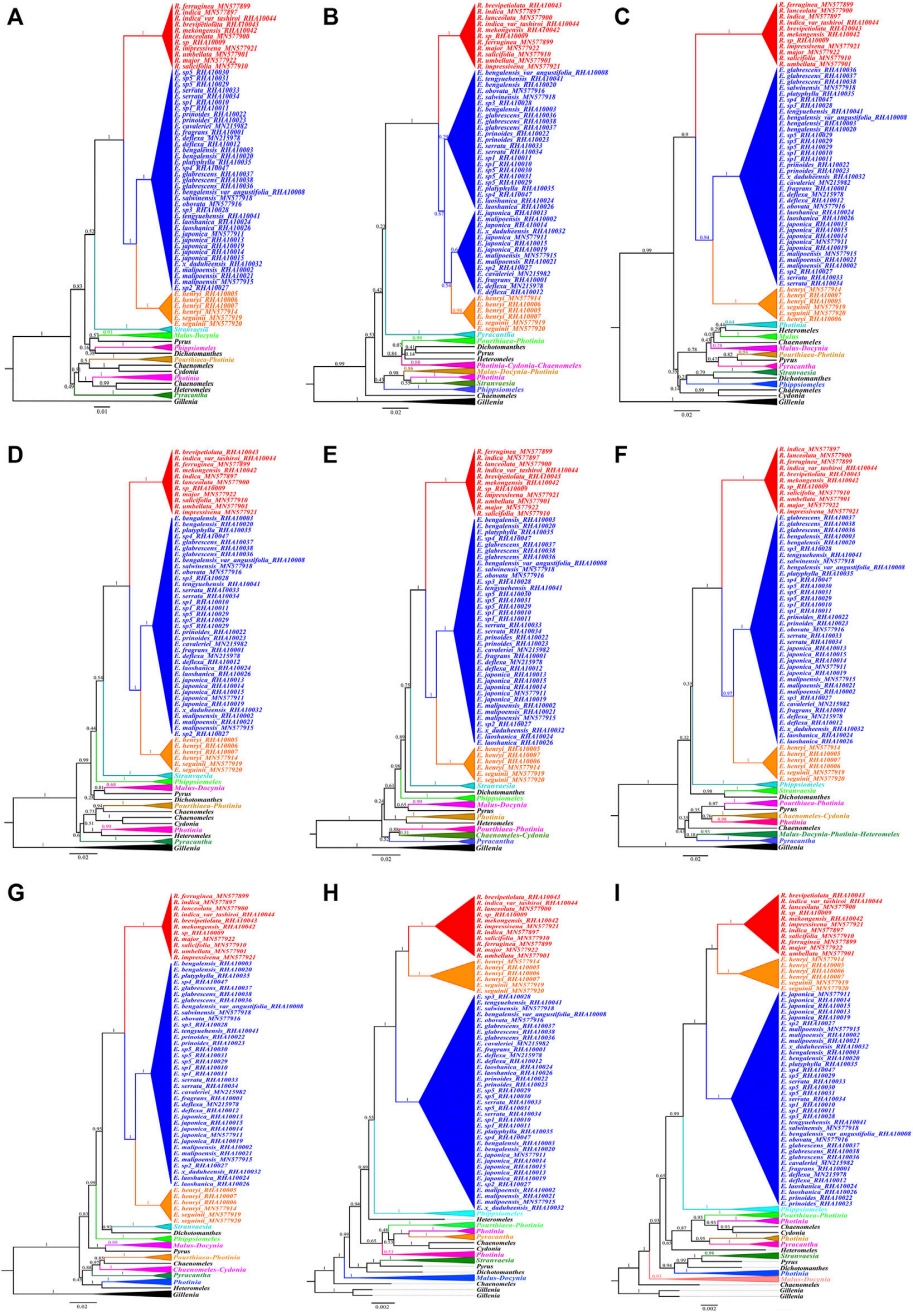
Bayesian inference trees of all the six gene spacer sequences datasets for 97 Rosaceae individuals: ETS **(A)**, ITS1 **(B)**, ITS2 **(C)**, ETS-ITS1 **(D)**, ETS-ITS2 **(E)**, ITS1-ITS2 **(F)**, ETS-ITS1-ITS2 **(G)**, 18S-5.8S-26S **(H)**, and 26S **(I)**. Numbers at each node are Bayesian posterior probabilities.

Incongruence is significant among the topologies obtained from the transcribed spacer regions and rRNA gene sequences. BI analyses of the ETS, ITS1, ITS2, ETS-ITS1, ETS-ITS2, ITS1-ITS2, and ETS-ITS1-ITS2 sequences fully resolved phylogenetic relationships among the major clades and most genera, and the *Eriobotrya* and *Rhaphiolepis* groups had high internal support, the exception being the data matrices of 18S-5.8S-26S and 26S (Figure 5). The phylogenetic analyses with 18S-5.8S-26S and 26S gene sequence found that two *Eriobotrya* species *E. henryi* and *E. seguinii* and all *Rhaphiolepis* species are located in the same clade, and other *Eriobotrya* species forming the next sister group, followed by *Phippsiomeles*.

## 4 Discussion

### 4.1 nrDNA Sequence Variation

Rapidly developing molecular markers, such as allozymes, DNA sequence including single nucleotide polymorphism (SNP), and simple DNA sequence repeated (SSR) loci have great potential in species identification, population structure analysis, and phylogenetic analysis. The standardized DNA regions include plastid *rbcL*, *matK*, and *trnH*-*psbA* and ribosomal DNA ITS1 or ITS2 (China Plant BOL Group et al., 2011). Among the 38 ITS1 sequences of *Eriobotrya* and *Rhaphiolepis* species, we manually identified mutation events including 28 SNPs and four SSR indels, and 61 SNPs, one SSR indel, and four non-SSR indels were accurately located in the ITS2 sequences. In addition, two non-SSR indels, four SSR indel, and 151 SNPs were found in the ETS regions, and four highly variable regions including ITS2, ETS, 26S, and ITS1 among the *Eriobotrya* and *Rhaphiolepis* species were identified. Both ITS1 and ITS2 regions were used to elucidate relationships among the taxa of *Eriobotrya* and *Rhaphiolepis* (Idrees et al., 2018; Idrees et al., 2020a; Idrees et al., 2020b; Kang et al., 2021). Here, two rarely reported highly variable loci ETS and 26S were present in *Eriobotrya* and *Rhaphiolepis* nrDNA sequence (Figures 2, 5). It was stressed that complementary ETS and 26S markers to the recommended ITS1 and ITS2 should continue to be assessed from nrDNA sequence. Through analysis of nrDNA sequences, additional plant ETS and 26S have been found and, in turn, have become valuable molecular markers for the identification of interspecific germplasm, which is helpful for the phylogeny relationship.

### 4.2 Relationship of *Eriobotrya* and *Rhaphiolepis*

All ML, BI, and MP analyses of the nrDNA sequences fully resolved phylogenetic relationships between *Eriobotrya* and *Rhaphiolepis* and confirmed that the monophyly of the *Eriobotrya* and *Rhaphiolepis*, respectively, in agreement with previously published phylogenetic relationships (Yang et al., 2012; Yang et al., 2017; Idrees et al., 2018; Idrees et al., 2020a; Idrees et al., 2020b; Kang et al., 2021). The topology obtained shows that nrDNA sequence, with appropriate sampling, can provide robust and significantly supported relationship among deep lineages of *Eriobotrya* and *Rhaphiolepis.* Seven such phylogenetically meaningful clades were identified among the deep lineages of the *Eriobotrya.* The backbones of the phylogenomic topologies obtained here are consistent with previously published phylogenetic relationships (Kang et al., 2021), but problems within several major clades in the *Eriobotrya* were solved. All the species in the genus *Rhaphiolepis* form a sister group of *Eriobotrya*, consistent with the study by Chen et al. (2020) and Kang et al. (2021). The Vietnamese species *E. condaoensis* is located in the earliest-diverging extant lineage within the *Eriobotrya*, which is in agreement with the previous phylogenetic results by Kang et al. (2021) who defined the relationships among 17 *Eriobotrya* species, respectively. This species is located in Con Dao National Park in southern Vietnam (Idrees et al., 2018), relatively far away from *E. henryi* and *E. seguinii* (Supplementary Figure S8). In the Clade 2, the sisterhood of *E. henryi* and *E. seguinii* is clarified, as found in previous studies (Idrees et al., 2020a; Idrees et al., 2020b; Liu et al., 2020; Kang et al., 2021). Previous phylogenetic analyses with plastid genome found that members of *E. laoshanica* were sister to *E. malipoensis* (Chen et al., 2020). However, our phylogenetic analyses show both species are located in different clades (Figures 3, 4). *E. laoshanica* is located in the Clade 4, while *E. malipoensis* is located in the Clade 3 with *E. grandiflora*, *E. hookeriana*, *E. japonica*, *E. petiolate*, *E.* sp2, and *E.* × *daduheensis*. *E. deflexa, E. cavaleriei* and *E. fragrans* are located in the Clade 5, likewise significant support in the ITS data (Kang et al., 2021) and the nuclear genes data (Chen et al., 2021) rather than the study of Idrees et al. (2020a); Idrees et al. (2020b), Chen et al. (2020), and Liu et al. (2020). In clade 6, *E. prinoides* is closely related to *E. elliptica* and *E.* serrata, but the relationship is not supported in the study of Idrees et al. (2020a); Idrees et al. (2020b); Kang et al. (2021), and Chen et al. (2021). Three Myanmar *Eriobotrya* species *E. glabrescens*, *E. platyphylla*, and *E. sp4*, were located in Clade 7 with six Chinese *Eriobotrya* species, *E. bengalensis*, *E. bengalensis* var*. angustifolia*, *E. obovate*, *E. salwinensis*, *E.* sp3, and *E. tengyuehensis* (Figures 3, 4). We further determined the relationships of 17 *Eriobotrya* and *Rhaphiolepis* species, *E.* elliptica, *E. glabrescens*, *E. laoshanica*, *E. platyphylla*, *E.* sp1, *E.* sp2, *E.* sp3, *E.* sp4, *E.* sp5, *E.* × *daduheensis*, *R. brevipetiolata*, *R. indica* var *tashiroi*, *R. mekongensis*, and *R.* sp.

Our nrDNA sequences of *Eriobotrya* and *Rhaphiolepis* yielded a fully resolved tree, consistent with the study of Kang et al. (2021), rather than that of Liu et al. (2020). The phylogenomic analysis showed *E. henryi* and *E. seguinii* is not nested among the members of *Rhaphiolepis*, which is incompatible with the chloroplast and nrDNA data in Liu et al. (2020). Liu et al. (2020) collected 16 taxa from *Eriobotrya* and *Rhaphiolepis*, and the molecular, morphological, and geographic evidence supported merging these two genera into one genus. In that research, the sampling proportions of *Eriobotrya* and *Rhaphiolepis* accounted for 27.6 and 70%, respectively, that is, only a small proportion of the sample was *Eriobotrya*. In addition, according to the distribution of *Eriobotrya* and *Rhaphiolepis*, no samples were collected from southeast Asia, southern Yunnan, Hainan, Sichuan, and Tibet. Among the 7 clades of *Eriobotrya* in our phylogenetic tree, Liu et al. (2020) only sampled species in clade 2, 3, 5, and 7. It is known that sample deviation could lead to phylogenetic errors, increasing the sampling of taxa is one of the most important ways to increase overall phylogenetic accuracy (Hillis, 1996; Hillis, 1998; Graybeal, 1998; Poe, 1998; Soltis et al., 1999; Pollock and Bruno, 2000; Pollock et al., 2000; Pollock et al., 2002; Murphy et al., 2001; Zwickl and Hillis, 2002). Research shows that increased taxon sampling provides new insights into the phylogeny and evolution of the subclass Calcaronea (Porifera, Calcarea) (Alvizu et al., 2018). In our research, adequate sampling of *Eriobotrya* species required sampling from Myanmar, Vietnam, Yunnan, Sichuan, Tibet, Hainan, and other places, greatly increasing the taxa and reducing the phylogenetic errors (Figure 1). In addition, we calculated the proportions of SNP mutations in the two genera of nrDNA. Among 23 species of *Eriobotrya* and 11 species of *Rhaphiolepis*, shared SNP sites accounted for 19.25% (Supplementary Figure S6A), whereas the shared sites accounted for 22.42% in the 8 species of *Eriobotrya* and 7 species of *Rhaphiolepis* (Supplementary Figure S6B). According to the distribution heat map of the two genera, although there are some overlapping areas, Eriobotrya species are mainly distributed in southwestern China and Indo-China (Supplementary Figure S7A), while Rhaphiolepis species are centered in southeastern China, and very scarcely recorded in Yunnan, Sichuan and Myanmar (Supplementary Figure S7B). Yunnan is the diversity center of *Eriobotrya* species, while the diversity center of *Rhaphiolepis* species is not.

The phylogenetic tree obtained with the 18S-5.8S-26S and 26S dataset showed a low resolution at all taxonomic levels, rendering most relationships inconclusive, which may be caused by the conservation of the rRNA gene functions. In analyzing the hypervariable regions of the two genera, we found that the mutation frequency was low in 18, 5.8, and 26S (Figure 2). Because of the conservatism of the rRNA gene sequencing, combined with a low mutation rate and limited information loci. In addition, there is an overlap between *E. henryi*, *E. seguinii* and *Rhaphiolepis* species, and hybridization may have occurred between *E. henryi*, *E. seguinii* and *Rhaphiolepis* species (Supplementary Figure S8). Resulting in the insertion of *E. henryi* and *E. seguinii* into the genus *Rhaphiolepis*. It is the same not only in plants but also in animals. This is due to the small number of informative sites in the18S rRNA. 18S proved to be highly conserved within Calcaronea and does not have sufficient signal to resolve phylogenetic relationships within the subclass (Alvizu et al., 2018).

### 4.3 Morphological Difference Between *Eriobotrya* and *Rhaphiolepis*

**FIGURE 6:**
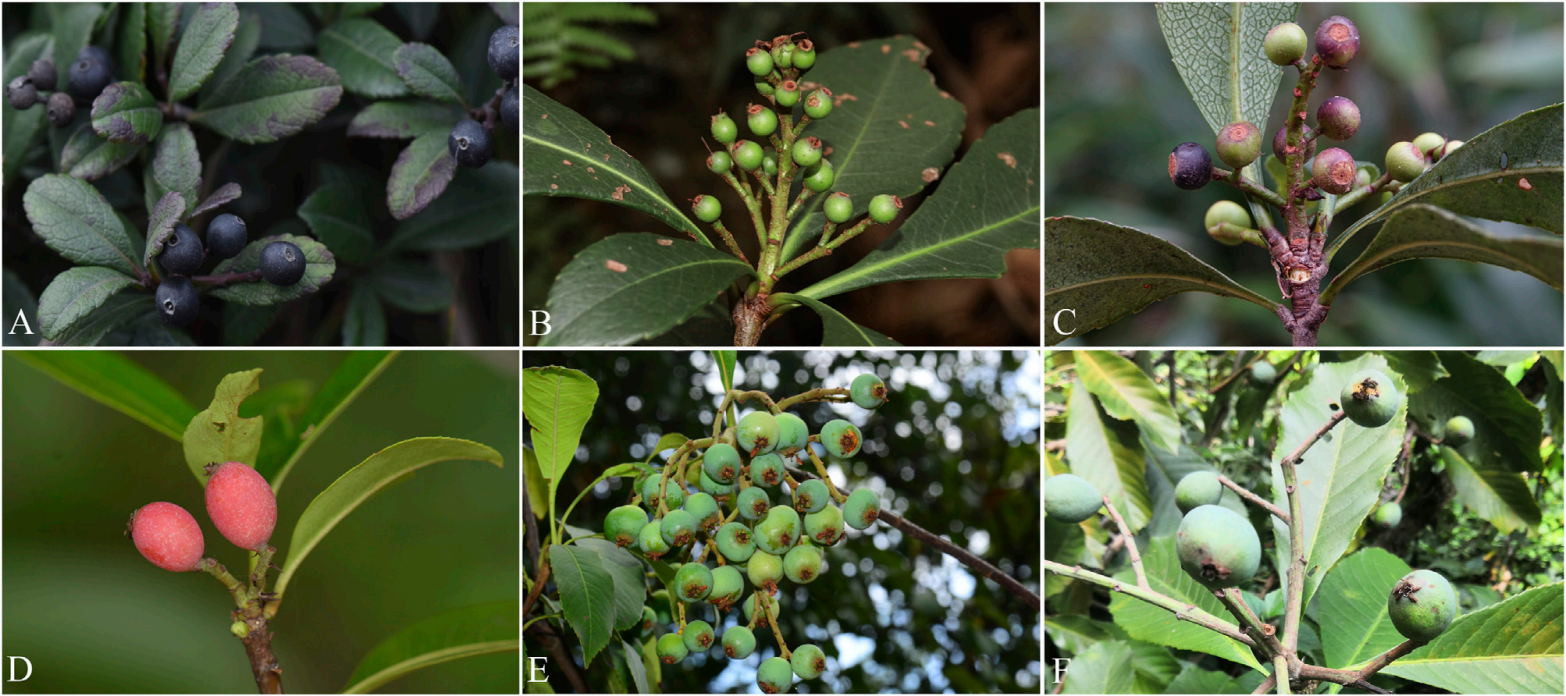
The caducous sepals—*Rhaphiolepis indica* **(A)**, *Rhaphiolepis major* **(B)**, *Rhaphiolepis umbellate* **(C)** and the persistent sepals—*Eriobotrya henryi* **(D)**, *Eriobotrya bengalensis* var. *angustifolia* **(E),** *Eriobotrya serrata* **(F)**.

In the taxonomic literature and flora, the persistence of sepals was used to distinguish between *Eriobotrya* and *Rhaphiolepis* (Vidal, 1968; Vidal, 1970; Kalkman, 1993, 2004; Lu et al., 2003). However, Liu et al. (2020) found that the sepals of *E. henryi* fell early in the field, and it was considered that the persistent sepal could not be used to distinguish between *Eriobotrya* and *Rhaphiolepis*. In addition, those authors argued that the camptodromous leaf venation in some loquat species of *Eriobotrya* and *Rhaphiolepis* lacked stability (Liu et al., 2020). However, taxonomic studies clearly show that both camptodromous and craspedodromous venation can be observed in *Eriobotrya* (Robertson et al., 1991; Gu and Spongberg, 2003.). We found that the sepals of *E. henryi* were persistent in the field (Figure 6D), and there was a significant difference between the two genera (Figure 6, Supplementary Table S5 and Supplementary Figure S9). Kang et al. (2021) reviewed the same picture and found that, although the *E. henryi* fruit calyx was caducous, it was intact in some photos. The (circular) annular ring after sepal senescence can only be observed in *Rhaphiolepis* (Robertson et al., 1991; Gu and Spongberg, 2003; Kang et al., 2021). The two genera can be well separated according to whether the sepals fall off and whether there is an annular ring after sepal senescence or not (Supplementary Table S5 and Supplementary Figure S9). In addition, Shaw (2020) stressed the importance of maintaining nomenclatural stability for *Eriobotrya* species with horticultural and agricultural value. Because of the conflicting issues we have found, we do not recommend *Eriobotrya* being incorporated into the genus *Rhaphiolepis*.

## Conclusion

Phylogenetic analysis of nrDNA sequences strongly supports *Eriobotrya* and *Rhaphiolepis* being monophyletic. In addition, phylogenetic analysis using nrDNA combined with ITS sequences, both the *Eriobotrya* and *Rhaphiolepis* were 100% supported monophyletic. Moreover, we speculate that the phylogenetic evidence for *Eriobotrya* as monophyly is congruent with the morphological characteristics of its leaves and the persistence of its sepals. It is not recommended that *Eriobotrya* be merged into *Rhaphiolepis*.

## Data Availability

The datasets presented in this study can be found in online repositories. The names of the repository/repositories and accession number(s) can be found in the article/Supplementary Material.

## Supplementary Material

Supplementary Figure S1 The patterns of nucleotide substitutions among *Eriobotrya* and *Rhaphiolepis*.

Supplementary Figure S2 The nrDNA sequences and each region SNP mutations in *Eriobotrya* and *Rhaphiolepis*.

Supplementary Figure S3 Molecular phylogenetic tree of 97 taxa of Rosaceae based on nrDNA sequences using maximum likelihood. Numbers at each node are the ML bootstrap support bootstrap support values.

Supplementary Figure S4 Molecular phylogenetic tree of 97 taxa of Rosaceae based on nrDNA sequences using maximum parsimony. Numbers at each node are the MP bootstrap support bootstrap support values.

Supplementary Figure S5 Molecular phylogenetic tree of 86 taxa of *Eriobotrya* and *Rhaphiolepis* and three related taxa of *Phippsiomeles*, based on the nrDNA sequence and ITS (containing only ITS1, 5.8S, and ITS2) sequences using maximum likelihood.

Supplementary Figure S6 Percentage of SNP mutations in the nrDNA 23 species of *Eriobotrya* and 11 species of *Rhaphiolepis* from this study **(A)** and 8 species of *Eriobotrya* and 7 species of *Rhaphiolepis* from Liu et al. (2020) **(B)**.

Supplementary Figure S7 Heat map of species distribution of *Eriobotrya* **(A)** and heat map of species distribution of *Rhaphiolepis* **(B)**.

Supplementary Figure S8 Species distribution map of *Eriobotrya* and *Rhaphiolepis*.

Supplementary Figure S9 Morphological character reconstruction of *Eriobotrya* and *Rhaphiolepis* on the Bayesian phylogenetic tree.

Supplementary Table S1 Sources of plant materials.

Supplementary Table S2 Plant materials for data matrix I.

Supplementary Table S3 Plant materials for data matrix II.

Supplementary Table S4 SNP mutations in nrDNA sequences of *Eriobotrya* and *Rhaphiolepis*.

Supplementary Table S5 Morphological comparisons amongst *Eriobotrya* and *Rhaphiolepis*.
