# Supplementary figures and images for: Increasing Taxa Sampling Provides New Insights on the Phylogenetic Relationship Between Eriobotrya and Rhaphiolepis
Source: Front Genet. 2022 Mar 15;13:831206. doi: 10.3389/fgene.2022.831206 (PMC8964991; doi:10.3389/fgene.2022.831206)

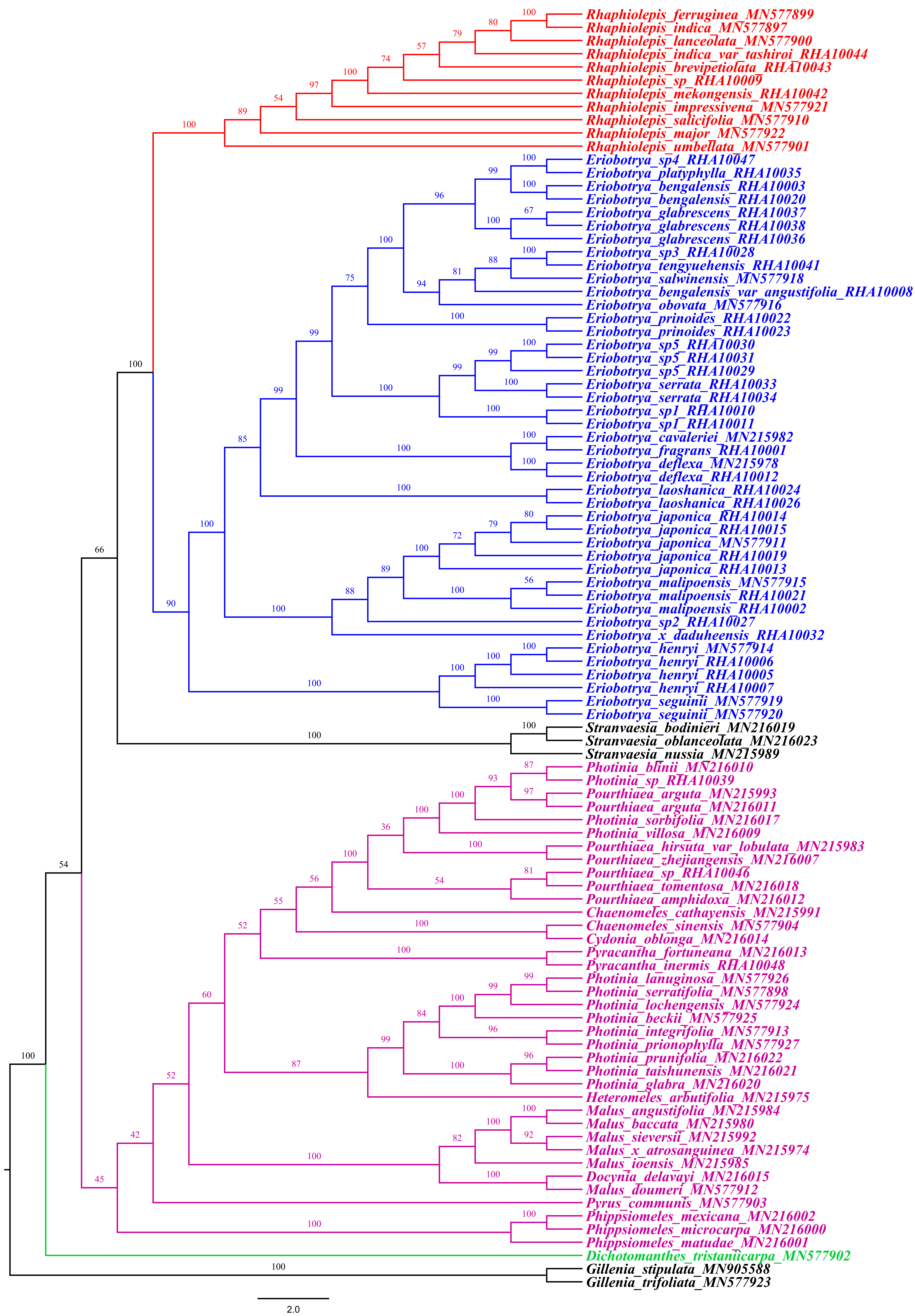

Supplement: Supplementary file 2 [file Image3.JPEG]

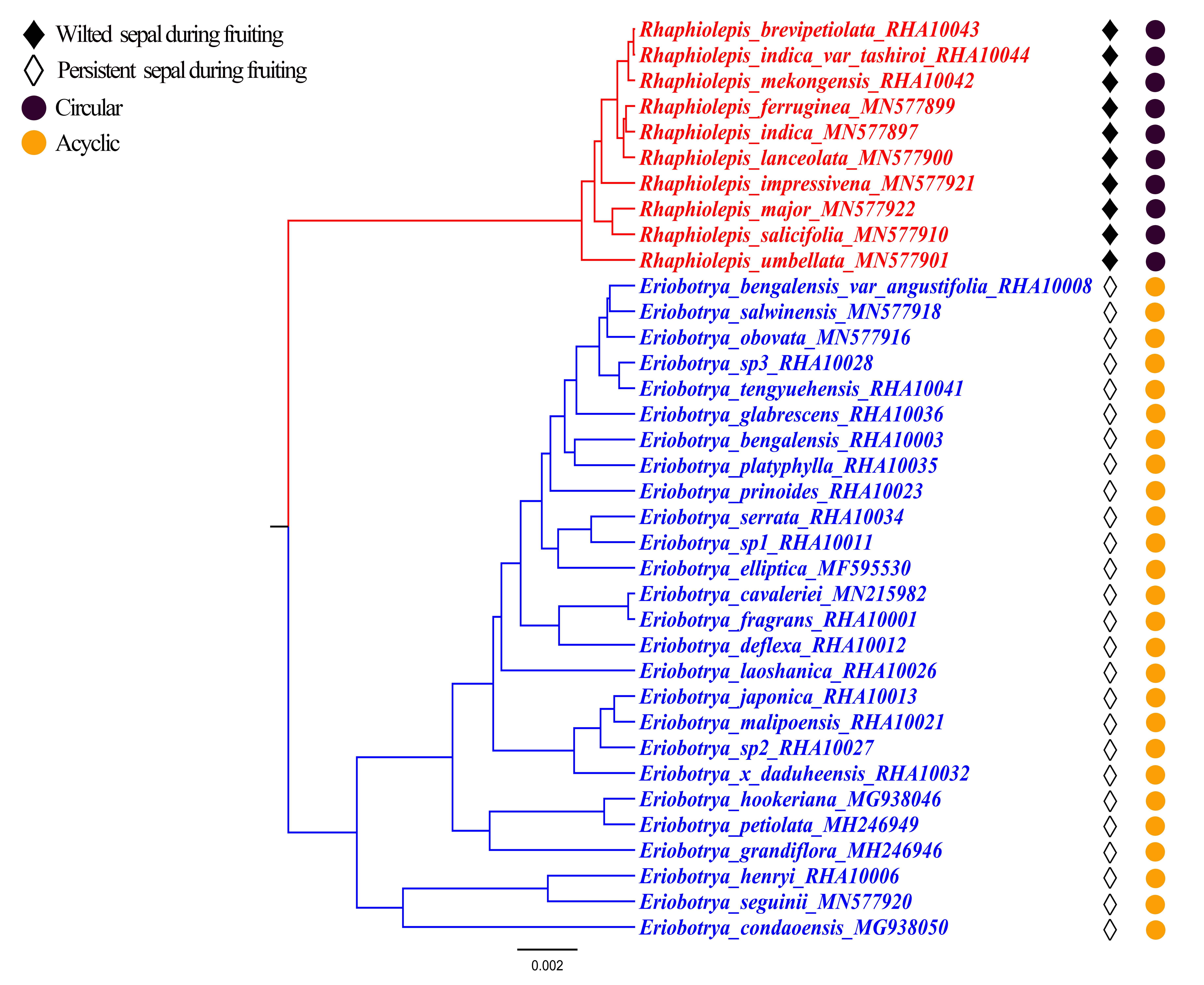

Supplement: Supplementary file 4 [file Image9.JPEG]

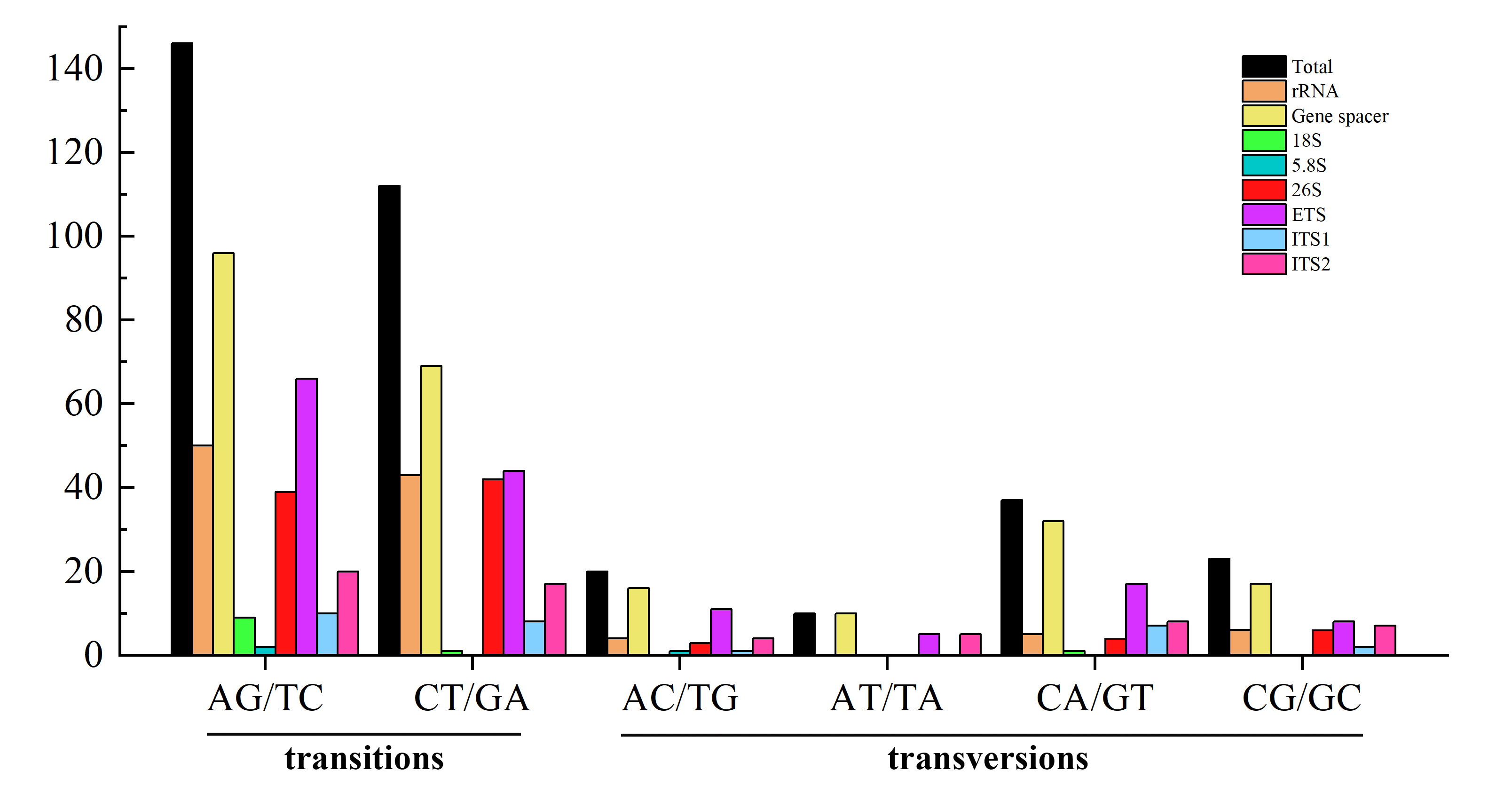

Supplement: Supplementary file 5 [file Image1.JPEG]

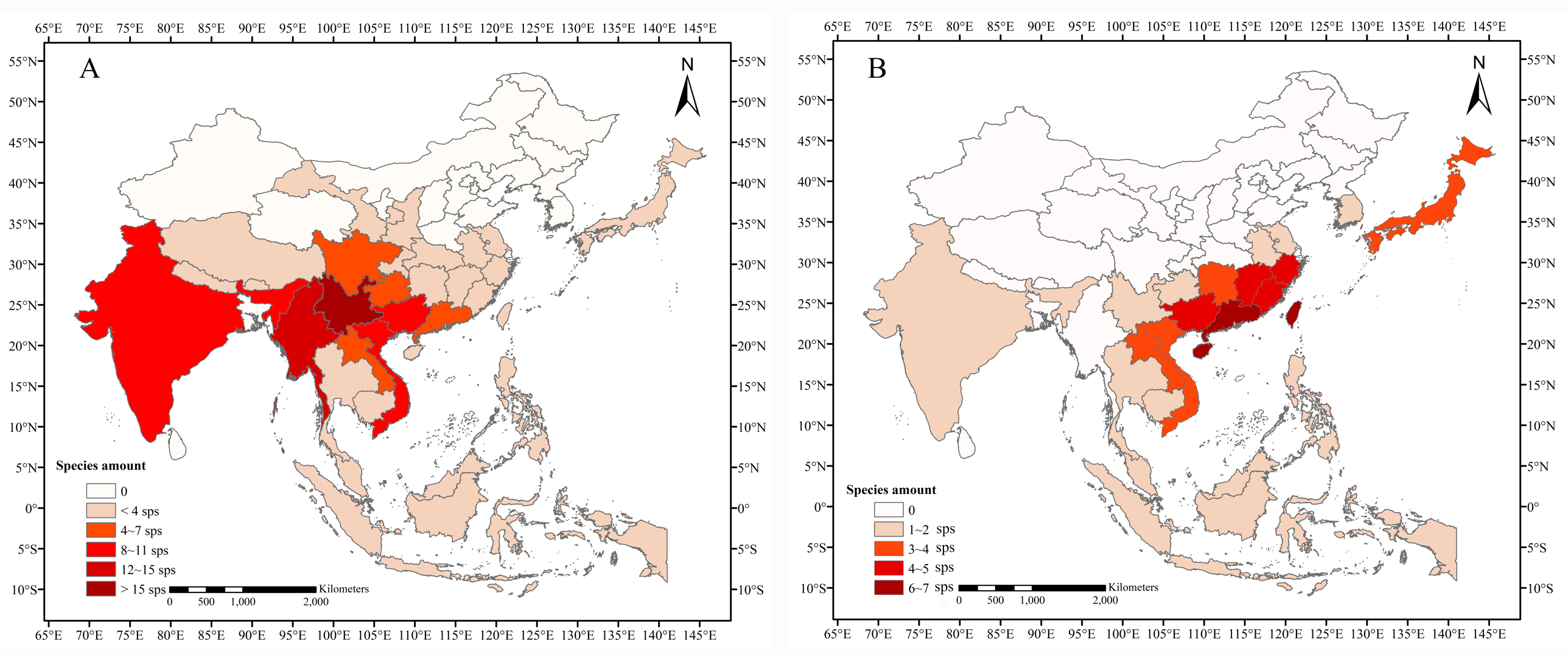

Supplement: Supplementary file 7 [file Image7.JPEG]

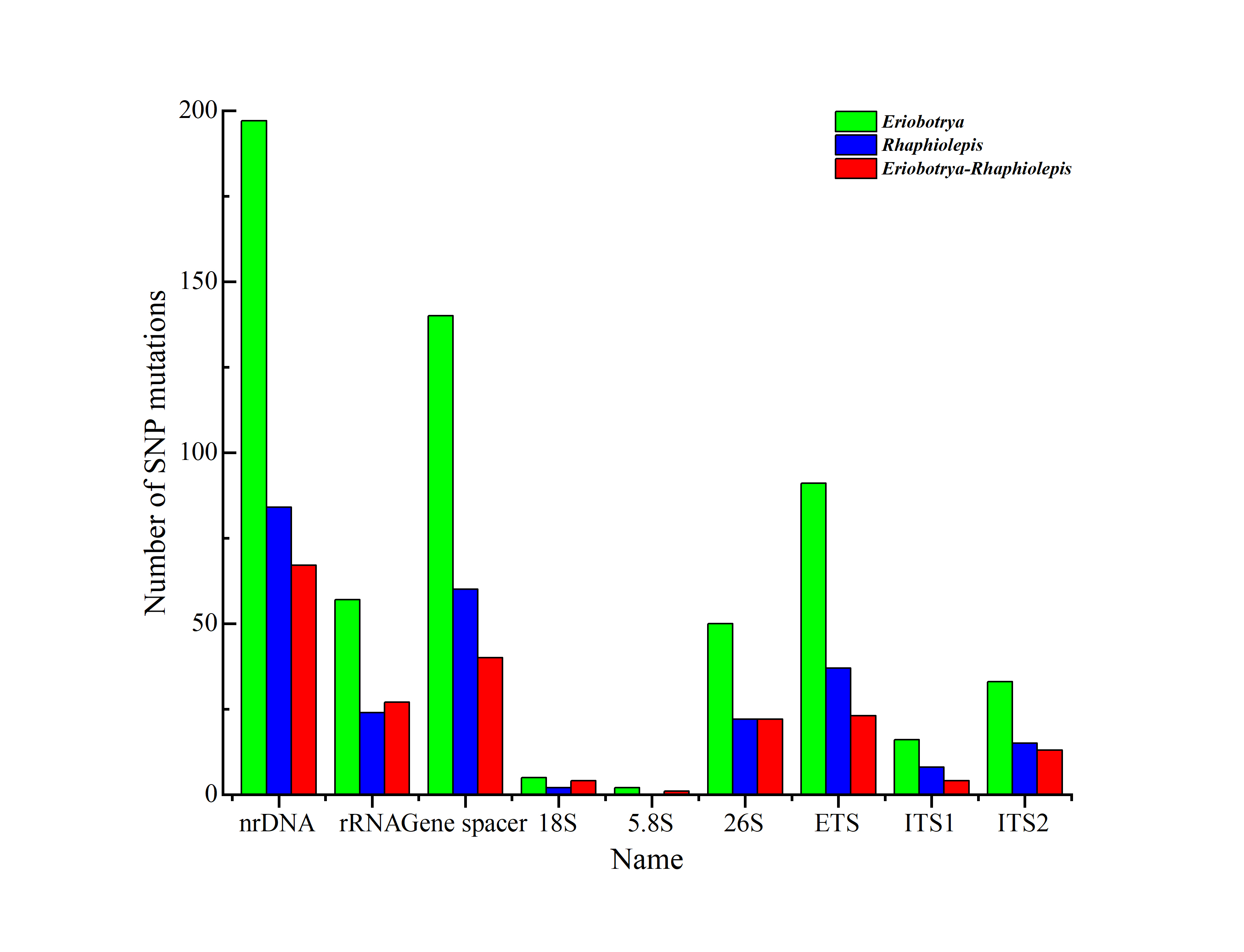

Supplement: Supplementary file 8 [file Image2.JPEG]

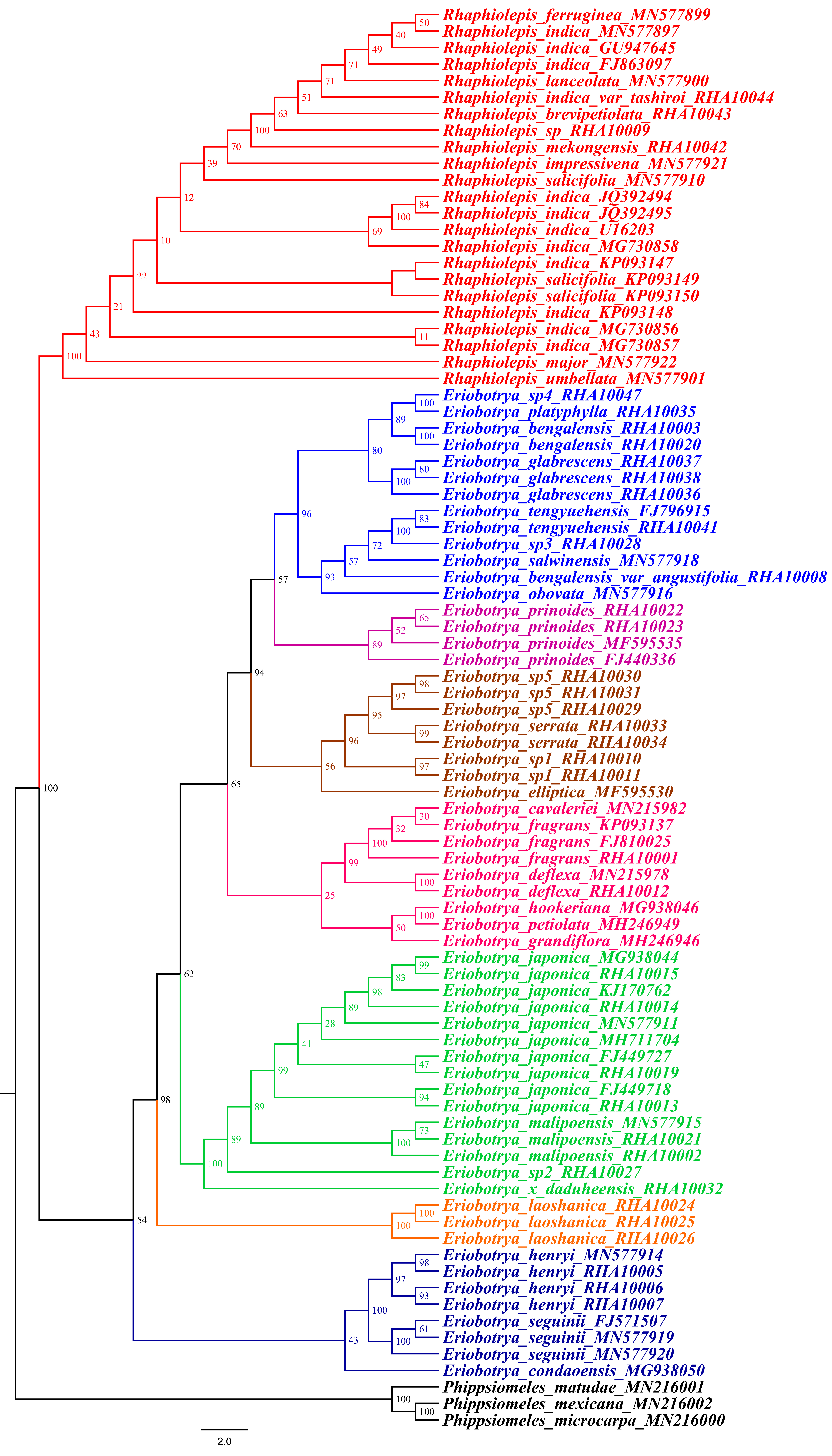

Supplement: Supplementary file 9 [file Image5.JPEG]

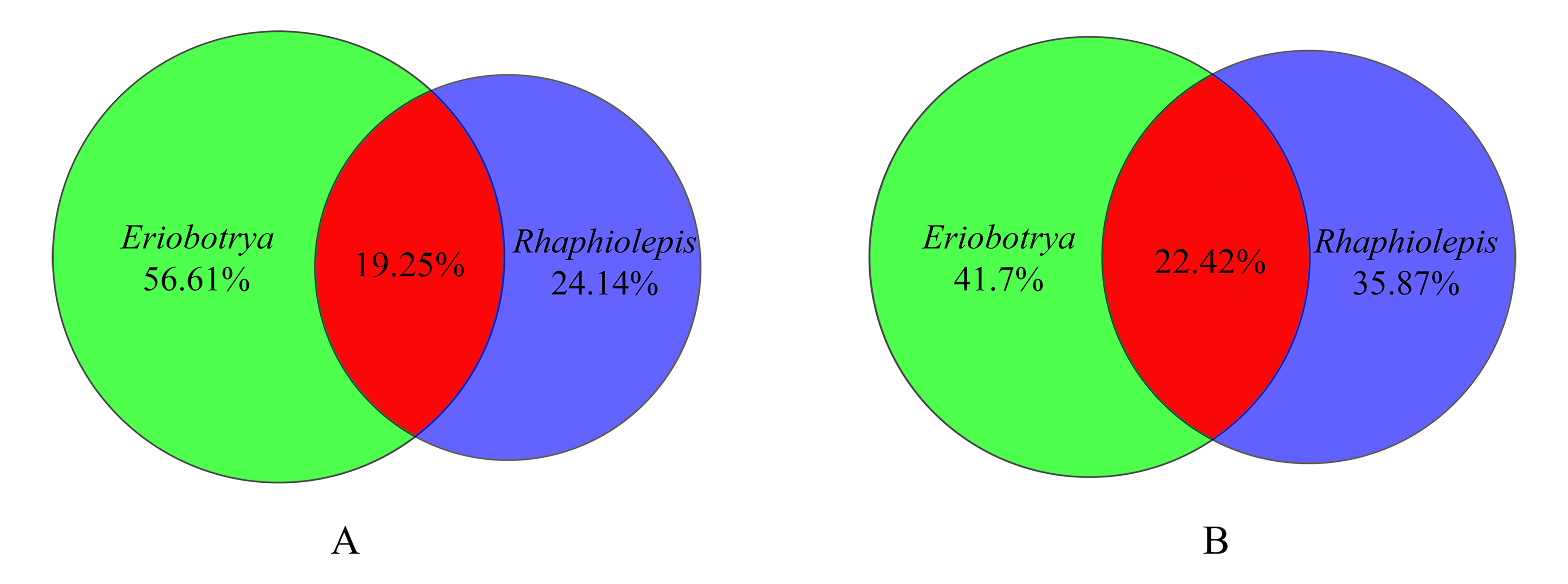

Supplement: Supplementary file 14 [file Image6.JPEG]
